# Adherence to cancer screening guidelines in Australian survivors of allogeneic blood and marrow transplantation (BMT)

**DOI:** 10.1002/cam4.729

**Published:** 2016-04-25

**Authors:** Gemma Dyer, Stephen R. Larsen, Nicole Gilroy, Lisa Brice, Matt Greenwood, Mark Hertzberg, Masura Kabir, Louisa Brown, Megan Hogg, Gillian Huang, John Moore, David Gottlieb, John Kwan, Jeff Tan, Christopher Ward, Ian Kerridge

**Affiliations:** ^1^ Northern Clinical SchoolFaculty of MedicineUniversity of SydneySydneyNSWAustralia; ^2^ Blood and Marrow Transplant NetworkNew South Wales Agency for Clinical InnovationSydneyNSWAustralia; ^3^ Institute of HaematologyRoyal Prince Alfred HospitalSydneyNSWAustralia; ^4^ Department of HaematologyRoyal North Shore HospitalSt. LeonardsNSWAustralia; ^5^ Northern Blood Research CentreKolling InstituteUniversity of SydneySydneyNSWAustralia; ^6^ Department of HaematologyPrince of Wales HospitalCamperdownNSWAustralia; ^7^ Westmead Breast Cancer InstituteWestmeadNSWAustralia; ^8^ Department of HaematologyCalvary Mater NewcastleNewcastleNSWAustralia; ^9^ Department of HaematoloyWestmead HospitalSydneyNSWAustralia; ^10^ Department of HaematologySt Vincents HospitalSydneyNSWAustralia

**Keywords:** Australia, blood and marrow transplant (BMT), cancer screening, late effects, secondary cancers, survivors

## Abstract

Allogeneic Blood and Marrow Transplant (BMT) survivors are at high risk of secondary cancers. Although current guidelines endorse survivors following Country‐specific general population screening recommendations to mitigate this risk, little is known about cancer screening adherence in Australian BMT survivors. We conducted a cross‐sectional survey of 441 BMT survivors who were >1 year post transplant, to explore rates of screening for secondary cancers and to identify barriers to cancer screening recommendations. Survey instruments included the Sydney Post‐BMT Survey, FACT‐BMT, DASS 21, The Chronic Graft versus Host Disease (GVHD) Activity Assessment–Patient Self‐Report (Form B), the Lee Chronic GVHD Symptom Scale, Fear of Cancer Recurrence Scale, and The Post Traumatic Growth Inventory. Fifty‐seven percent of respondents were male, median age 54 years, and 40% were >6 years post‐BMT. Rates of cancer screening adherence were as follows: cervical 63.4%, breast 53.3%, skin 52.4%, and bowel 32.3%. Older BMT survivors and those >2 years post transplant were more likely to undergo cancer screening. Improved quality of life was associated with screening for skin, breast, and cervical cancer. Fear of cancer recurrence negatively impacted on cervical screening. For those who had not undergone screening, the majority reported not being advised to do so by their treatment team. This study is the largest and most comprehensive to date exploring cancer screening adherence in BMT survivors in Australia. These data provide the basis for health service reform to better meet the needs of BMT survivors and provide evidence to support counseling and education of both patients and professionals.

## Introduction

Survivors of allogeneic Blood and Marrow Transplant (BMT) are at a significant risk of developing many long‐term and adverse late effects in the years following transplantation 1. Of these late effects, secondary malignancies are a particular concern. Cumulative incidence rates of up to 12% at 15 years post‐BMT have been reported, and no plateau has been identified 2, 3. All cancers have been found to occur in survivors of BMT with skin, thyroid, oral cavity, esophagus, breast, liver, brain/nervous system, bone, and connective tissue cancers, all more frequently diagnosed in BMT survivors than the general population 4, 5, 6. Risk factors for higher rates of secondary cancers include younger age at BMT, total body irradiation (TBI), prolonged immunosuppression, chronic graft‐versus‐host disease (cGVHD), and smoking prior to allogeneic BMT 5, 6, 7, 8.

For almost a decade international consensus guidelines for the care of long‐term survivors of BMT have been available 9, 10. These guidelines include recommendations for cancer screening, preventive health care and health promotion, noting that survivors follow general population screening recommendations in their country for breast, cervical, skin, genital, and bowel cancer, and avoid high‐risk behaviors (smoking, excess drinking, overweight and obesity, inactivity, and unprotected skin UV exposure). These guidelines also make clear that in patients with chronic GVHD, additional attention needs to be paid to surveillance for oral, pharyngeal, and early skin cancer. While some controversy exists regarding the commencement, frequency, and modality for breast cancer screening, it is generally suggested that for woman who received TBI and/or chest irradiation, mammography screening should be commenced at age 25 or 8 years after radiation exposure, whichever occurs later, but no later than age 40 years 8, 10, 11.

In Australia, the Commonwealth Government funds three national screening programs to reduce the burden of cancer nationwide; Breast Screen Australia, the National Cervical Screening Program, and the National Bowel Cancer Screening Program. These programs offer free screening to the Australian public at specific age and interval time points (Table 1) 12, 13. Cancer Australia (the lead national cancer control agency) also advocates known healthy lifestyle behaviors such as quitting smoking, being ‘sun smart’, being active, maintaining a healthy diet, and limiting alcohol intake for the entire population. Participation in these programs and health promotion behaviors in Australian BMT survivors is largely unknown.

**Table 1:** Australia's National Cancer Screening Programs with recommendations for the general population 13

Cancer Screening Program 13	Recommendations 13
BreastScreen Australia	BreastScreen Australia invites women aged 50–74 to have free 2 yearly mammogram. Women aged 40–49 and 75 and over are eligible to receive free mammograms, but do not receive an invitation to attend.
National Bowel Cancer Screening Program (NBCSP)	The NBCSP invites men and women turning 50, 55, 60, 64, 65, 70, 72, and 74 to screen for bowel cancer. Participants are sent a free, easy to use screening kit that can be completed at home. Between 2015 and 2020, more age groups will be added to the screening program: 2017—68, 58, and 54 year olds. 2018—62 and 66 year olds. 2019 and 2020—52 and 56 year olds.
National Cervical Screening Program (NCSP)	The NCSP invites all women aged between 18 and 70 who have ever been sexually active to have 2 yearly Pap tests. Cervical screening is provided through general practice, community or women's health centers, family planning clinics, sexual health clinics, or Aboriginal Medical Services. From 1 May 2017, the NCSP will be changed to inviting women aged 25–74 years (both HPV vaccinated and unvaccinated) to undertake an HPV test every 5 years.

Despite the availability of long‐term follow‐up guidelines 9, 10, and the excess burden of secondary cancers post allogeneic BMT 14, international studies have shown that cancer screening uptake and health behaviors in BMT survivors are similar, if not worse than people who have had cancer but not had an allogeneic BMT, and people who have never had cancer 15, 16. In this study we aimed to explore rates of screening for secondary malignancies in an Australian cohort of allogeneic BMT survivors and identify barriers to adherence with cancer screening recommendations.

## Methods

A cross‐sectional survey of BMT survivors was undertaken to explore late effects of BMT and the quality of survival post transplant. This survey of BMT survivors in New South Wales (NSW) Australia included questions regarding rates of secondary cancers, adherence to cancer screening, and modifiable healthy lifestyle behavior, together with demographic and social characteristic associated with barriers to uptake of cancer screening recommendations.

NSW is Australia's most populous state with a population of ~7.5 million and covers an area of 800,628 km^2^. Over a third of residents live outside the greater Sydney area 17. At the time of study commencement there were four adult allogeneic transplant centers in NSW, all based in Sydney and collectively performing approximately 175 BMTs annually 18.

Potential participants were identified from allogeneic transplant databases from all adult allogeneic transplant centers in NSW. Participants were eligible if they were >18 years of age and had undergone an allogeneic BMT between 1 January 2000 and 31 December 2012, could read and write English, and could provide consent. Names and phone numbers were provided to the research team. Consenting participants were given the option to self‐complete the questionnaire or complete it via a phone interview with one of the researchers. A second round of telephone calls was made to 187 participants who had not returned the survey within a month. All authors had access to primary clinical trial data. The study protocol was approved by the Northern Sydney Local Health District Human Research Ethics Committee (NSLHD Reference: 1207‐217M).

## Instruments

Participants were asked to complete seven instruments.

*The Sydney Post BMT Study Survey (SPBS)* was developed by the research team from a review of the literature and discussion with patients attending BMT long‐term follow‐up clinics. The survey comprised 402 questions grouped into 20 domains and included questions relating to secondary cancer diagnosis, cancer screening adherence, and lifestyle behavior choices. Other relevant domains included demographics, medical complications, tests and assessments, medications and therapies, infections, vaccinations, complementary therapy use, relationship status, income (Australian Dollars, AUD), and lifestyle factors, following allogeneic BMT. The questionnaire used tick box responses, short answer questions, and five‐step Likert scales measuring attitudes and other factors and took approximately 1 h to complete. The questionnaire was piloted with six BMT survivors in clinic and phone interviews to assess face and content validity and to check for comprehension. For each consenting participant, data were collected on dates of diagnosis and transplant, stage/remission status at transplant, transplant conditioning, GVHD prophylaxis, stem cell source, and donor type.

Cancer screening adherence and health behavior choices were analyzed according to a range of demographic, transplant, psychosocial, and lifestyle variables assessed using the *Functional Assessment of Cancer Therapy – Bone Marrow Transplant (FACT‐BMT Version 4)* 19, 20, anxiety stress and depression (*The DASS 21)* 21, 22, 23, chronic GVHD (*The Chronic GVHD Activity Assessment – Patient Self Report (Form B)* 24, *The Lee Chronic GVHD Symptom Scale)* 25, the *Fear of Cancer Recurrence (FoCR) Scale* 26, and *The Post Traumatic Growth Inventory (PTGI)* score 27, 28. For ease of completion all instruments were combined into one booklet.

### Statistical analysis

Categorical responses were summarized using frequencies and percentages. Parametric continuous variables were summarized using means and standard deviations, and nonparametric variables using medians, interquartile ranges (IQR), or ranges. Odds ratios and 95% confidence limits, Pearson *χ* ^2^ test, or Fishers Exact tests were used for comparative analysis of dichotomous categorical variables and multivariable logistic regression to adjust for relevant confounders. Two sample comparisons of parametric and nonparametric data were determined using the independent *t*‐test, and Wilcoxon rank sum tests, respectively; greater than two‐sample comparisons were determined using one‐way Analysis of Variance (ANOVA) and Kruskal–Wallis tests, respectively. A two‐tailed *P*‐value <0.05 was used as the level of statistical significance.

Statistical analysis was performed using STATA version 12.1 statistical package (StataCorp, College Station, TX).

## Results

A total of 1475 Allogeneic BMT were performed in the study period. Of the 669 recipients known to be alive at study sampling, 583 were contactable and were sent study packs. Four hundred and forty one (66% of total eligible, 76% of those contacted) returned the completed survey. Three percent (17) declined participation.

Of those completing the survey, 250 (57%) were male and 191 (43%) female. The median age of survey respondents was 54 years (Range: 19–79). The median age at time of transplant was 49 years (Range: 17–71) (Table 2).

**Table 2:** Participant characteristics

Sociodemographic	
Gender (*n* = 441)	
Male *n* (%)	250 (56.7%)
Female *n* (%)	191 (43.3%)
Age (years) at survey (*n* = 441)	
Median (IQR; range)	54 (44,62; 19–79)
Age (years) at transplant (*n* = 441)	
Median (IQR; range)	49 (38, 56; 17–71)
Ethnicity (*n* = 372)	
Caucasian, European *n* (%)	323 (86.8%)
Other *n* (%)	49 (13.2%
Educational status (*n* = 333)	
University (some/completed) *n* (%)	154 (46.2%)
Other^a^ *n* (%)	179 (53.8%)
Post transplant income status (AUD^d^) (*n* = 423)	
Low income $20,000–$39,999 *n* (%)	155 (36.6%)
Middle income $40,000–$79,999 *n* (%)	123(29.1%)
High income ≥ $80,000 *n* (%)	145 (34.3%)
Residence (*n* = 431)	
Major city *n* (%)	311 (72.2%)
Other (inner regional, outer regional, remote) *n* (%)	120 (27.8%)
Relationship status (*n* = 434)	
Married–Defacto *n* (%)	344 (79.3%)
Other (separated, single, divorced) *n* (%)	90 (21.8%)
Transplant –related	
Years since transplant (*n* = 441)	
<2 years *n* (%)	58 (13.1%)
=2 to <6 years *n* (%)	204 (46.3%)
=6 to <10 years *n* (%)	117 (26.5%)
≥10 years *n* (%)	62(14.1%)
Underlying diagnosis (*n* = 423)	
Acute leukemia (AML/ALL) *n* (%)	226 (53.4%)
Other^b^ *n* (%)	197 (46.6%)
Remission status (*n* = 405)	
First/second complete remission	271 (66.9%)
Other^c^	134 (33.1%)
Donor type (*n* = 441)	
Sibling *n* (%)	250 (59.9%)
Matched unrelated *n* (%)	158 (36.0%)
Haploidentical *n* (%)	10 (2.3%)
Mismatched unrelated *n* (%)	21 (4.8%)
Stem cell source(*n* = 441)	
Bone marrow *n* (%)	48 (10.9%)
Peripheral blood *n* (%)	381 (86.4%)
Cord blood *n* (%)	12 (2.7%)
Conditioning chemotherapy (*n* = 439)	
Myeloablative	214 (48.7%)
Reduced intensity	225 (51.3%)

a Other Education—secondary school (some or complete); trade or diploma.

b Other diagnoses: CML, Chronic Myeloid Leukemia; CLL, Chronic Lymphocytic Leukemia; NHL, Non‐Hodgkin Lymphoma; HL, Hodgkin Lymphoma; MM, Multiple Myeloma; Myelodysplastic Syndrome/Myeloproliferative disorder; Other (unspecified).

c Other remission status; more than second complete remission; Refractory; Chronic Phase; Accelerated Phase; Blast Crisis; Partial Remission; other (unspecified).

d AUD—Australian Dollars; IRQ, interquartile ranges.

### Secondary cancer diagnosis

One hundred and six (24.0%) reported a diagnosis of at least one cancer following BMT of which 104 were nonrelapse malignancies. Skin cancers accounted for the largest number of secondary cancers (Table 3).

**Table 3:** Secondary cancer diagnosis post blood and marrow transplant (BMT)

Cancer types (*N *= Number of Responses)	*n* (% reporting cancer type of total responses)
Skin cancer (*n* = 404)	93(23.0%)
Skin cancer type	% of all skin cancers
Basal cell carcinoma (BCC)	41 (44%)
Squamous cell carcinoma (SCC)	14 (15%)
Melanoma	5 (6%)
Mixed	17 (18%)
BCC + SCC	14(15%)
BCC + Melanoma	2 (2%)
SCC + Melanoma	1(1%)
Unspecified/don't know	16(17%)
Mouth cancers (*n* = 392)	6 (1.5%)
Other (*n* = 370)	18 (4.9%)*n* (% of all other cancers)
Urological (prostate and/or bladder)	5/18 (27%)^a^
Breast	2(11%)
Bowel	1(6%)
Ovarian	1(6%)
Myeloid sarcoma	1(6%)
Head (unspecified)	1(6%)
Hematological (nonrelapse)	5 (27%)^b^
Hematological (relapse)	2(11%)^c^

a 3 prostate, 1 bladder, 1 bladder + prostate.

b 1 NHL (primary = AML); 1 NHL (Primary = SAA); 2 Hodgkin Lymphoma (Primary = NHL); 1 post transplant lymphoproliferative disease.

c 1 Relapse AML; 1 relapse Mantle Cell Lymphoma; AUD, Australian Dollars

### Cancer screening

#### Skin cancer screening

A total of 436 (98.9%) participants provided a response to whether or not they had had undergone skin cancer screening since transplant. Two hundred and twenty eight (52.3%) reported having had a skin check and 208 (47.7%) reported never having had a skin check since BMT. Of those who reported having had a skin check, 75% had done so in the preceding 18 months (range 1 month to 9 years). One hundred and sixty six of the 228 (72.8%) reported attending for skin checks at least once a year.

Demographic, social, transplant‐related, treatment‐related, and behavioral factors were assessed for their association with having skin checks as part of cancer screening post transplant. Of note, skin checks were not significantly associated with skin GVHD, receipt of azole antifungals, or outdoor occupations (gardening, construction, or agriculture). Univariate analysis demonstrated a significantly increased odds of skin checks with older age, higher education status, being in a married or defacto relationship, and a high compliance with “sun smart” behaviors including the routine use of sunscreen, hats, sun protective attire, sunglasses, and sun avoidance during the daily periods for peak exposure. Factors associated with a reduced odds of skin checks on univariate analysis included an acute leukemia diagnosis, receipt of a myeloablative conditioning regimen, and being within 2 years of transplant. After adjusting for potential confounders, those factors that demonstrated an independent and significant association with having skin checks post transplant included older age (Adjusted 1.03 95% CI: 1.0, 1.05; *P* = 0.03), higher educational status (Adjusted Odds Ratio [AOR]: 1.87 95% CI: 1.11, 3.15; *P* = 0.02), and “Sun smart” behavior (AOR: 1.89 95% CI: 1.06, 3.37; *P* = 0.03).

Compliance with skin cancer screening was further assessed against measures including quality of life (FACT‐BMT and subscales) and psychological morbidity (DASS21 and subscales), Lee GVHD scores, self‐reported GVHD symptom severity, and Fear of Cancer recurrence and Post Transplant Growth Inventory scores. Survivors who had skin checks had significantly higher scores on FACT emotional subscale (*P* = 0.03), BMT subscale (*P* = 0.007), and overall FACT‐BMT scores (*P* = 0.03), and significantly lower scores on depression subscales (*P* = 0.02), with no significant difference observed on other subscale or composite DASS21 measures (Appendix A1).

Reasons cited for not undergoing skin cancer screening in 208 patients included lack of time in 13 (6.2%), cost in five (2.5%), and belief that screening was not necessary in 54 (26.0%). One hundred and forty nine patients (71.6%) indicated that they had not been advised by their treating team to undergo skin cancer screening. Twenty nine (13.9%) of those who had never undertaken skin cancer screening were receiving azole antifungal therapy.

#### Bowel cancer screening

A total of 432 participants provided a response to whether or not they had undergone bowel cancer screening (either colonoscopy or stool hemoccult testing) since transplant. One hundred and forty (32.4%) reported having had a bowel cancer check and 292 (67.6%) reported not having had a bowel cancer check since BMT. Of those who reported having had a bowel cancer check, 75% had done so in the preceding 2 years (range <1 month to 11 years). Forty‐seven of 140 (33.8%) reported having bowel checks at least every 2 years.

On univariate analysis, older BMT survivors and those in a married or defacto relationship showed a significantly increased odds of undergoing bowel cancer screening. Transplant‐related factors including an underlying diagnosis of acute leukemia and receiving myeloablative conditioning were associated with significantly decreased odds of bowel cancer screening. On multivariable analysis, the only variable with an independent and significant increased association with bowel cancer screening was older age (AOR: 1.06; 95% CI: 1.03, 1.08; *P* < 0.0001).

No significant differences were evident in DASS21 and FACT‐BMT scores and subscales, Lee GVHD or other psychosocial metrics in those who reported bowel screening and those who did not (Appendix A2).

Of the 292 patients who did not have bowel screening, 8 (2.7%) cited time, 2 (0.7%) cost, and 75 (25.7%) feeling that screening was not necessary as the main reasons for not attending to a bowel check since transplant. Two hundred and twenty five patients (77% of those not having a bowel cancer check) reported that they had not been advised to undergo bowel cancer screening by their treating team.

#### Cervical cancer screening

A total of 186 of female participants provided a response to whether or not they had had a Paapaniolou (pap) smear since transplant. One hundred and eighteen (63.4%) females reported having had a pap smear and sixty eight (36.6%) reported not having had a pap smear since BMT. Of those who reported having had a pap smear, 75% had done so in the preceding 2 years (range: 1 month to 5 years).

Younger age was significantly associated with having had a pap smear (*P* = 0.04) and women who were less likely to have had a pap smear if within 2 years of the transplant procedure. Following multivariable analysis, a trend for a reduced odds with older age was observed (AOR: 0.97; 95% CI: 0.94, 1.0: *P* = 0.09) and a significantly reduced odds of pap screening for women less than 2 years post transplant (AOR: 0.30; 95% CI: 0.11, 0.85: *P* = 0.02).

Those reporting cervical cancer screening post transplant showed no overall differences in DASS 21 scores, although on a trend toward lower Anxiety scores was observed in females who had undergone pap screening (*P* = 0.06). Patients undergoing pap screening reported a trend toward higher emotional subscale scores (*P* = 0.054) and significantly higher functional well‐being (*P* = 0.008) and overall scores on FACT‐BMT (*P* = 0.02). This would suggest a positive association between improved quality of life in women who had pap screening. Lower uptake of cervical screening was associated with a significantly increased fear of cancer recurrence (FoCR) score (*P* = 0.003). (Appendix A3).

Barriers to undergoing cervical cancer screening included lack of time in 8 (11.8%), cost in 2 (2.9%), and a belief that Pap screening was not necessary in 20 (29.4%). A total of 31 women (45.6%) reported that they had not been advised to have a Pap smear by their treating team.

#### Breast cancer screening

A total of 184 female participants provided a response to whether or not they had had a mammogram for breast cancer screening since transplant. Ninety‐eight (53.3%) females reported having had a mammogram and 86 (46.7%) reported not having had a mammogram since BMT. Seventy‐five percent reporting having a mammogram in the preceding 2 years (range 2.5 month to 4 years). The age of first mammogram was reported by 68 women; in their 20s (8), 30s (12), 40s (31), 50s (16), and 60s (1). Older age (AOR: 1.11; 95% CI: 1.07, 1.16; *P* < 0.001) and residing in a city/inner‐regional center (AOR: 5.33; 95% CI: 1.37, 20.8; *P* = 0.03) were the only variables associated with a significantly increased odds of screening mammography on multivariable analysis. Total Body Irradiation (TBI) as part of the conditioning regimen showed a trend toward increased mammography uptake (AOR: 2.35; 95% CI: 0.99, 5.58; *P* = 0.052) and being less than 2 years post transplant a trend toward decreased mammography uptake (AOR: 0.31; 95% CI: 0.09, 1.05; *P* = 0.06).

For those reporting mammography screening post transplant, there were no overall differences in DASS 21 scores, although lower depression subscales were associated with mammography uptake (*P* = 0.04). Patients undergoing mammography reported significantly higher emotional (*P* < 0.001) and BMT subscale scores on FACT‐BMT (*P* = 0.02). This would suggest a positive association between improved quality of life in women who had mammography. Mammography screening was associated with significantly lower median Lee GVHD severity scores (*P* = 0.02), although no significant differences were observed using the alternative metric for GVHD severity (cGVHD activity assessment Form B) (Appendix A4).

For those not having mammography, 5 (5.8%) reported lack of time, 2 (2.3%) had an issue with cost, 23 (26.7%) felt it was not necessary, and 57 (66.3%) reported not being advised by their treating team to undergo breast cancer screening.

## Discussion

The results of this study confirm that secondary cancers occur commonly after allogeneic transplantation 7, 29, 30 and that cancer screening is not being performed according to recommended BMT long‐term follow‐up guidelines 10, 15 or with recommendations for cancer screening in the general Australian population 12. Our cohort had lower rates of screening for bowel cancer (32.3%), cervical cancer (63.4%), and breast cancer (53.3%) than previous studies in BMT survivor populations 31. These rates are, however, similar to adherence rates among the general Australian population who participate in Breast Screen Australia (55%), the National Cervical Screening Program (58%), and the National Bowel Cancer Screening Program (33%) 12. Only half of the cohort had had a skin cancer check (52.4%) following transplant. This is significant because although there is currently insufficient evidence to support population‐based screening for nonmelanocytic and melanoma skin cancer 32, and skin cancer screening not recommended by major public health bodies 12, 32, Australians experience a melanoma incidence rate 11 times that of the average world rate 12 and BMT recipients are at markedly higher risk of developing all forms of skin cancer as a consequence of cutaneous graft‐versus‐host disease (GVHD), long‐term use of immunosuppressive drugs, and azole antifungal agents 29.

In this study the major determinant of cancer screening, with the exception of cervical screening, was older age. In contrast to other studies, which have shown that being further out from BMT decreased adherence to preventive care practices 31, we observed a trend toward increased screening for skin, bowel, and breast cancers in late compared to early transplant survivors. A significant association with cervical screening was observed in females beyond the first 2 years of their transplant procedure. It is difficult, however, to know the significance of this finding as there are no data regarding whether the age time points for general population cancer screening apply to BMT survivors, many of whom experience an increased risk from a younger age of secondary cancer, particularly breast and skin cancers. For this reason alone the ‘benchmarking’ of cancer screening adherence against general population recommendations raise real questions regarding best practice and the possibility of both over‐ and underdiagnosis 33. In this regard it is noteworthy that rates of cancer screening in our study population were not only inconsistent with recommendations for general population screening but also with recommendations for BMT recipients and for high‐risk cancer survivors. The finding that women exposed to TBI were no more likely to have mammography or to commence mammography at an earlier age is of enormous concern given the recognized association between radiation exposure and breast cancer 34.

Interestingly, our study found a significant association between participation in skin, cervical, and breast cancer screening and higher quality of life. Although this is reassuring to those involved in post transplant care, it runs counter to recent literature that suggests that cancer screening can increase anxiety and overdiagnosis 33, 35. There are at least two possible explanations for this finding—firstly, that those who report higher quality of life may be more motivated to maintain good health, or conversely, participating in cancer prevention may confer quality of life and survival benefits.

We were unable to identify a significant association between social factors such as being in a married/defacto relationship with increased screening uptake, a finding that is otherwise well‐described in other cancer screening studies 36, 37, 38, 39.

Fear of cancer recurrence (FoCR) in those with an underlying hematological malignancy at transplantation was investigated for its associations with screening uptake. FoCR was negatively associated with cervical screening uptake, but had no association with other screening procedures. Positive measures of personal growth assessed using the PGTI was not associated with adherence to cancer screening. The severity of GVHD symptoms was further explored for any potential association with screening uptake. We hypothesized that more symptomatic GVHD may result in patients being under closer medical surveillance and, therefore, more likely to be screened for cancers. Lee GVHD scores were observed to in fact be lower in females who did undergo post transplant breast screening, which may also attest to the better quality of life that such patients have.

Perhaps most importantly, the results of our study provide clues as why BMT survivors may not participate in cancer screening programs. Over a quarter of our respondents reported that they did not feel that screening was necessary—suggesting failures in the education and/or counseling of transplant recipients—a finding reported in previous studies 14. In addition, of those who reported that they had not undergone any cancer screening, the vast majority gave the reason that the screening test/s had not been recommended by their treating team. While we did not verify this report for individual patients, it is consistent with the findings of studies conducted in general populations 40 and may likely be true in this population given the inadequate resourcing of post transplant care in many BMT centers. Cost was reported to be a barrier to uptake for very few survivors in our study; however, financial difficulties (low income) have been shown to impact cancer screening rates in other populations 38. This may be a function of Australia's aforementioned free national cancer screening programs. The barrier of cost was reported for younger survivors who do not meet age criteria (e.g., annual mammograms for those age 25 years if they had TBI). These explanations for the low rates of adherence with cancer screening recommendations are also consistent with the literature that suggests that many factors may act as barriers to optimal preventive care including: lack of knowledge regarding the importance of cancer screening in both patients and providers, deficiencies in the organization of preventive health‐care services 31, and skepticism regarding the value of screening 33, 41.

This study is important because it is one of the largest studies describing adherence with cancer screening guidelines in a BMT population and the first to explore cancer risk behavior and adherence to cancer screening guidelines in an Australian cohort of BMT survivors. Although the sample size and high response rate (76%) make it likely that these results represent an accurate account of BMT survivor's health behaviors, there are a number of limitations to our study which may limit the generalizability of these results to BMT survivors in other countries and other settings. These limitations are principally a function of our study population and include Australia's geographical size, population pattern, climate and health system (which includes both universal publicly funded and private health care), and funded national cancer screening programs. The fact that the study relied upon self‐reporting and did not capture data on nonresponders also limits our findings. Another area which may have been of interest is that relating to digital rectal examination (DRE) and/or prostate‐specific antigen (PSA) for prostate cancer screening in BMT survivors, however, due to the controversy regarding these modalities 42, 43, 44, 45, and that general population prostate cancer screening is not recommended in Australia, we did not ask participants about this. Additionally, it should be noted that as only two of the respondents had a recurrence of the malignancy for which they were transplanted, our findings only apply to survivors who remain disease free following BMT. Also, as we did not ask respondents about adherence to cancer screening guidelines pre‐BMT, we are not able to make any correlation between pre‐ and post‐BMT practices.

What this study makes clear is that recommendations for cancer screening and for preventive health‐care post‐BMT are, in many situations, not being followed by health‐care services and/or adopted by the target population. Although the exact reasons for this require further qualitative study, it seems likely that this is a result of both systems failures and inadequate or unsuccessful patient education. It is also possible, but entirely speculative, that this may result from awareness that there is currently limited data to support cancer screening in BMT patients—including those most at risk. Absence of good quality long‐term data does not, however, create an argument for therapeutic nihilism or for failures to deliver comprehensive care post‐BMT. Rather, data from studies such as this one should be used to drive the development and implementation of models of chronic care post‐BMT that address gaps in health promotion, behavior modification, and cancer screening in order to prevent morbidity and mortality in long‐term BMT survivors and increase awareness in health professionals and patients alike of the increased risk of secondary cancers in survivors of BMT.

## Conflict of Interest

None declared.

## Appendix A1. Demographic, social determinants, transplant factors, and instrument measures (QoL, psychological morbidity, GVHD, FoCR, PTGI), and their associations with having skin cancer screening.

**Table 4:**

	Skin check*N* = 228	No skin check*N* = 208	OR (95% CI)	Adjusted OR(95% CI)^a^ *P*‐value
Gender				
Male	127/228 (55.7%)	121/208 (58.2%)	0.90 (0.61, 1.34)	
Female	101/228 (44.3%)	87/208 (41.8%)	0.61	
Age (median, IQR)	58 (48, 64)	51 (42, 59)	1.03 (1.02, 1.05)	1.03 (1.0, 1.05)
			<0.0001	*P* = 0.03
Residence				
City/inner regional	209/223 (93.7%)	182/203 (89.7%)	1.72 (0.81, 3.77)	
Outer regional/remote	14/223 (6.3%)	21/203 (10.3%)	0.13	
Education				
Some/completed university	91/173 (52.6%)	60/156 (38.5%)	1.77 (1.12, 2.82)	1.87 (1.11, 3.15)
Trade/some or complete secondary school	82/173 (47.4%)	96/156 (61.5%)	0.03	*P* = 0.02
Household income (AUD)				
Low income $20,000–$39,999	71 (32.7%)	83 (41.3%)	0.69 (0.45, 1.05)	0.73 (0.42, 1.27)
Middle/high income ≥ $40,000	146 (67.3%)	118 (58.7%)	0.07	*P* = 0.27
Marital status				
Married/Defacto	188/225 (83.6%)	152/205 (74.1%)	1.77 (1.08, 2.92)	0.80(0.42, 1.51)
Single/divorced/separated	37/225 (17.3%)	53/205 (25.8%)	0.02	*P* = 0.48
Occupation				
Gardener	11/210 (5.2%)	1/187 (0.5%)		
Building/construction	17/211 (8.1%)	13/189 (6.9%)		
Agriculture/Farm worker	9/209 (4.3%)	7/189 (3.7%)	1.44 (0.77, 2.79)	
			0.22	
Any outdoor occupation				
Yes (Gardener, Builder, Ag worker)	31/215 (14.4%)	20/192 (10.4%)		
No	184/215 (85.6%)	172/192 (89.6%)		
Years since transplant				
<2 years	20/228 (8.8%)	37/208 (17.8%)	0.44 (0.23, 0.82)	0.69 (0.32, 1.48)
≥2 years	208/228 (91.2%)	171/208 (82.2%)	0.005	*P* = 0.34
Underlying diagnosis	100/215 (46.5%)	123/203 (60.6%)	0.56 (0.38, 0.85)	
Acute leukemia	115/215 (53.5%)	80/123 (39.4%)	0.004	0.70 (0.42, 1.16)
Other				*P* = 0.17
Donor type				
Matched (sibling/unrelated)	213/226 (94.2%)	190/208 (91.3%)	1.55 (0.70, 3.54)	
Mismatched (haploidentical, unrelated)	13/226 (5.8%)	18/208 (8.7%)	0.24	
Conditioning				
Myeloablative	98/226 (43.4%)	115/208 (55.3%)	0.62 (0.42, 0.92)	0.85 (0.49, 1.48)
Reduced Intensity	128/226 (56.6%)	93/208 (44.7%)	0.01	*P* = 0.57
Self‐reported skin GVHD				
Yes	113/228 (49.5%)	89/208 (42.8%)	1.31 (0.88, 1.95)	
No	115/228 (50.4%)	119/208 (57.2%)	0.16	
Medications				
Immunosuppression				
Yes	73/228 (32.0%)	82/208 (39.4%)	0.72 (0.48, 1.09)	
No	155/228 (68.0%)	126/208 (60.6%)	0.11	
Azole antifungals				
Yes	24/228 (10.5%)	29/208 (13.9%)	0.73 (0.39, 1.34)	
No	204/228 (89.5%)	179/208 (86.1%)	0.27	
Routine use of sun protection				
Yes	183/223 (82.1%)	147/203 (72.4%)	1.74 (1.07, 2.84)	**1.89 (1.06, 3.37)**
No	40/223 (17.9%)	56/203 (27.6%)	0.02	*P* ** = 0.03**
DASS 21 score (median, IQR)	18 (8, 38)	20 (10,42)		*P* = 0.2
Depression subscale	4 (0,12)	6 (2,14)		*P* ** = 0.03**
Anxiety subscale	4 (2,10)	4 (2,12)		*P* = 0.53
Stress subscale	8 (2,16)	8 (4,16)		*P* = 0.69
FACT‐BMT score (median, IQR)	112 (96, 125)	106 (88,119)		*P* ** = 0.01**
Physical well‐being subscale	24 (20, 27)	24 (19,26)		*P* = 0.09
Social well‐being subscale	22 (17,25)	20 (15, 24)		*P* = 0.11
Emotional well‐being subscale	17 (15, 19)	16 (14,19)		*P* ** = 0.03**
Functional well‐being subscale	21 (16,24)	19 (14,24)		*P* = 0.08
BMT well‐being subscale	29 (25,33)	27 (23,32)		*P* ** = 0.007**
LEE GVHD score (Median, IQR)	21 (9,32)	19 (10, 29)		*P* = 0.51
Global severity GVHD symptoms (%)				
None	23 (16.1%)	16 (13.3%)		*P* = 0.64
Mild	65 (45.5%)	64 (53.3%)		
Moderate	39 (27.3%)	29 (24.2%)		
Severe	16 (11.2%)	11 (9.2%)		
Fear of cancer recurrence (median, IQR)	13 (10,16)	14 (11, 17)		*P* = 0.22
Post transplant growth inventory score	57 (37, 70)	60 (44,70)		*P* = 0.42

AUD, Australian Dollars; IRQ, interquartile ranges; PTGI, The Post Traumatic Growth Inventory.

a Variables included in multivariable logistic regression model to adjust for confounding: age, education, income and marital status, time from transplant (<2 years compared to later), underlying diagnosis (acute leukemia compared to other), conditioning regimen (myeloablative compared to reduced intensity), and “sun smart” practices.

Bold text indicates statistically significant figures.

## Appendix A2. Demographic, social determinants, transplant factors, and instrument measures (QoL, psychological morbidity, GVHD, FoCR, PTGI), and their associations with having bowel cancer screening.

**Table 5:**

	Bowel Ca screen	No Bowel Ca Screen	OR (95% CI)	Adjusted OR (95% CI)
	*N* = 140	*N* = 292	*P*‐value	*P*‐value^a^
Gender				
Male	79/140 (56.4%)	168/292(57.5%)	0.95(0.62, 1.47)	
Female	61/140 (43.6%)	124/292 (42.5%)	0.83	
Age (Median, IQR)	59(53, 64)	50(40, 60)	1.06(1.03, 1.08)	**1.06 (1.03,1.08)**
			<0.0001	*P* ** < 0.0001**
Residence				
City/inner regional	128/137(93.4%)	262/285(91.9%)	1.25(0.54, 3.15)	
Outer regional/remote	9/137(6.6%)	23/285(8.1%)	0.69	
Education				
Some/completed University	50/105(47.6%)	101/222(45.5%)	1.09(0.66,.78)	
Trade/some or complete secondary school	55/105(52.4%)	121/222(54.5%)	0.72	
Household income (AUD)				
Low income $20,000–$39,999	56/136(41.2%)	95/280(33.9%)	1.36(0.87, 2.12)	
Middle/High income ≥ $40,000	80/136 (58.8%)	185/280(66.1%)	0.15	
Marital status				
Married/Defacto	116/136(85.3%)	222/289(76.8%)	1.75(0.99, 3.20)	1.14(0.63, 2.07)
Single/Divorced/separated	20/136(14.7%)	67/289(23.2%)	0.04	*P* = 0.65
Years since transplant				
<2 years	13/140(9.3%)	44/292(15.1%)	0.58(0.27, 1.14)	0.61(0.30, 1.21)
≥2 years	127/140(90.7%)	248/292(84.9%)	0.13	*P* = 0.16
Underlying diagnosis				
Acute Leukemia	60/135 (44.4%)	158/279(56.6%)	0.61(0.39, 0.95)	0.75(0.48, 1.19)
Other	75/135(55.6%)	121/279 (43.4%)	0.02	*P* = 0.23
Donor type				
Matched (sibling/unrelated)	126/138(91.3%)	274/292(93.8%)	0.69(0.30, 1.62)	
Mismatched (haploidentical, unrelated)	12/138	18/292(6.2%)	0.42	
Conditioning				
Myeloablative	57/138(41.3%)	155/292(53.1%)	0.62(0.40, 0.95)	1.20(0.72,2.00)
Reduced Intensity	81/138(58.7%)	137/292(46.9%)	0.02	*P* = 0.49
GVHD				
Yes	98/135(72.6%)	196/290(67.6%)	1.27(0.79, 2.06)	
No	37/135(27.4%)	94/290(32.4%)	0.3	
Self‐reported GUT GVHD				
Yes	26/140 (18.6%)	34/292 (11.6%)	1.73(0.95, 3.12)	1.66(0.90, 3.05
No	114/140(81.4%)	258/292(88.4%)	0.05	*P* = 0.10
Medications				
Immunosuppression				
Yes	49/140(35.0%)	103/292 (35.3%)	0.98(0.63, 1.54)	
No	91/140(65.0%)	189/292(64.7%)	0.95	
DASS 21 score (Median, IQR)	19(10,38)	20(9,40)		*P* = 0.97
Depression subscale	4(2,14)	6(2,14)		*P* = 0.79
Anxiety subscale	4(2,8)	6(2,10)		*P* = 0.43
Stress subscale	10(4,18)	8(2,16)		*P* = 0.43
FACT‐BMT score(Median, IQR)	112(93, 122)	108(92,122)		*P* = 0.39
Physical well‐being subscale	24(20, 26)	24(19,26)		*P* = 0.46
Social well‐being subscale	22(17,25)	21(16, 24)		*P* = 0.14
Emotional well‐being subscale	17(14, 19)	17(14,19)		*P* = 0.79
Functional well‐being subscale	20(15,24)	20(15,25)		*P* = 0.83
BMT subscale	29(24,33)	28(23,32)		*P* = 0.51
LEE GVHD score (median, IQR)	16(8, 28)	21(10,32)		*P* = 0.06
Global severity GVHD symptoms (%)				
Mild	15(16.5%)	26 (15.0%)		*P* = 0.76
Moderate	47(51.6%)	81 (46.8%)		
Severe	20(22.0%)	48 (27.7%)		
Very Severe	9(9.9%)	18(10.4%)		
Fear of cancer recurrence (Median, IQR)	**13(10, 15)**	**14(10,17)**		*P* ** = 0.3**
Post transplant growth inventory Score	58(42, 70)	58(38, 71)		*P* = 0.81

AUD, Australian Dollars; IRQ, interquartile ranges; PTGI, The Post Traumatic Growth Inventory

a Variables included in multivariable logistic regression model to adjust for confounding: age, marital status, time from transplant (<2 years compared to later), underlying diagnosis (acute leukemia compared to other), conditioning regimen (myeloablative compared to reduced intensity), and gut GVHD.

Bold text indicates statistically significant figures.

## Appendix A3. Demographic, social determinants, transplant factors, and instrument measures (QoL, psychological morbidity, GVHD, FoCR, PTGI), and their associations with cervical cancer screening

**Table 6:**

	Cervical screen *N* = 118	No cervical screen *N* = 68	OR (95% CI)	Adjusted OR (95% CI)^a^
				*P*‐value
Age (Median, IQR)	50(42, 58)	56 (42, 63)	0.04	0.97(0.94, 1.00)
				*P* = 0.09
Residence				
City/inner regional	109/117(93.2%)	58/65(89.2%)	1.64(0.48, 5.47)	
Outer regional/remote	8/117(6.8%)	7/65(10.8%)	0.4	
Education				
Some/completed University	41/93 (44.1%)	19/54 (35.2%)	1.45(0.69, 3.09)	
Trade/some or complete secondary school	52/93(55.9%)	35/54 (64.8%)	0.3	
Household income (AUD)				
Low income $20,000–$39,999	45/113 (39.8%)	25/66(37.9%)	1.81(0.86, 3.93)	1.13(0.58,2.19)
Middle/High income ≥ $40,000	68/113(60.2%)	41/66(62.1%)	0.09	*P* = 0.71
Marital status				
Married/Defacto	92/113 (81.4%)	52/87 (77.6%)	1.26(0.55, 2.82)	
Single/Divorced/separated	21/113 (18.6%)	15/67(22.4%)	0.54	
Years since transplant				
<2 years	8/118 (6.8%)	14/68 (20.6%)	0.28(0.09, 0.77)	**0.30(0.11, 0.85)**
≥2 years	110/118 (93.2%)	54/68(79.4%)	0.008	*P* ** = 0.02**
Underlying diagnosis				
Acute Leukemia	71/111(64.0%)	39/66(59.1%)	1.22(0.62, 2.40)	
Other	40/111(36.0%)	27/66(40.9%)	0.52	
Donor type				
Matched (sibling/unrelated)	108/118(91.5%)	64/68(94.1%)	0.67(0.15, 2.47)	
Mismatched (haploidentical, unrelated)	10/118(8.5%)	4/68(5.9%)	0.58	
Conditioning				
Myeloablative	70/118(59.3%)	33/68(48.5%)	1.55(0.81, 2.95)	0.98(0.47, 2.03)
Reduced Intensity	48/118(40.7%)	35/68(51.5%)	0.15	*P* = 0.97
GVHD				
Yes	78/117(66.7%)	44/66(66.7%)	1.0(0.50, 1.98)	
No	39/117(33.3%)	22/66(33.3%)	1	
Self‐reported vaginal GVHD				
Yes	25/118(21.2%)	16/68(25.5%)	0.87(0.40, 1.92)	
No	93/118(78.8%)	52/68(76.5%)	0.71	
Medications				
Immunosuppression				
Yes	25/118 (21.2%)	21/68(30.9%)	0.60(0.30, 1.26)	0.78(0.38, 1.61)
No	93/118 (78.8%)	47/68(69.1%)	0.14	*P* = 0.50
DASS 21 score (Median, IQR)	18(8,34)	20(10,40)		*P* = 0.21
Depression subscale	4(1,9)	4(0,12)		*P* = 0.28
Anxiety subscale	4(1,8)	6(2, 10)		*P* = 0.06
Stress subscale	8(2,14)	10(4,16)		*P* = 0.63
FACT‐BMT score (Median, IQR)	111(99, 123)	104(91, 119)		*P* = 0.02
Physical well‐being subscale	25(22,27)	24(18,26)		*P* = 0.12
Social well‐being subscale	22(17,26)	21(18,26)		*P* = 0.89
Emotional well‐being subscale	17(15,19)	16(14, 18)		*P* = 0.054
Functional well‐being subscale	21(18,26)	19(16,23)		*P* ** = 0.008**
BMT well‐being subscale	28(25,32)	27(22, 32)		*P* = 0.07
LEE GVHD score (Median, IQR)	14 (8, 28)	19(9, 28)		*P* = 0.25
Global severity GVHD symptoms (%)				
Mild	12(16.0%)	6(17.1%)		
Moderate	41(54.5%)	15(42.9%)		*P* = 0.68
Severe	19(25.3%)	12(34.3%)		
Very Severe	3(4.0%)	2(5.7%)		
Fear of cancer Recurrence (Median, IQR)	12(9,15)	15(10,18)		*P* ** = 0.003**
Post transplant Growth Inventory Score	59(44,71)	68(49, 82)		*P* = 0.1

PTGI, The Post Traumatic Growth Inventory.

a Potential confounders included in multivariable logistic regression: age, income status (low compared to middle/high income), early post transplant (within 2 years), conditioning regimen (myeloablative compared to reduced intensity), and taking immunosuppression (tacrolimus, cyclosporine, mycophenolate, or prednisolone). IRQ, interquartile ranges.

Bold text indicates statistically significant figures.

## Appendix A4. Demographic, social determinants, transplant factors, and instrument measures (QoL, psychological morbidity, GVHD, FoCR, and PTGI), and their associations with breast cancer screening.

**Table 7:**

	Mammogram *N* = 98	No Mammogram *N* = 86	OR (95% CI) *P*‐value	Adjusted^a^ OR (95% CI) *P* **‐**value
Age (median, IQR)	57(50, 63)	43(32, 54)	1.09 (1.06, 1.13)	1.12(1.08, 1.17)
			<0.0001	*P* ** < 0.0001**
Residence				
City/inner regional	92/96 (95.8%)	73/84(86.9%)	3.46(0.97, 15.4)	**4.81(1.16, 19.9)**
Outer regional/remote	4/96 (4.2%)	11/84 (13.1%)	0.06	*P* ** = 0.03**
Education				
Some/completed University	35/83(42.2%)	23/63(36.5%)	1.27(0.61, 2.63)	
Trade/some or complete secondary school	48/83(57.8%)	40/63(63.5%)	0.49	
Household income (AUD)				
Low income $20,000–$39,999	39/96 (40.6%)	30/82(36.6%)	1.18(0.62, 2.28)	
Middle/High income ≥ $40,000	57/96 (59.4%)	52/82(63.4%)	0.58	
Marital status				
Married/Defacto	80/94(85.1%)	62/84(73.8%)	2.03(0.90, 4.64)	1.13(0.42, 3.07)
Single/divorced/separated	62/94 (73.8%)	22/84(26.2%)	0.06	*P* = 0.63
Years since transplant				
**<**2 years	6/98 (6.1%)	14/86 (16.3%)	0.33(0.10, 0.99)	0.31(0.09, 1.05)
≥2 years	92/98 (93.9%)	72/86(83.7%)	0.03	*P* = 0.06
Underlying diagnosis				
Acute Leukemia	57/96(59.4%)	53/81(65.4%)	0.77(0.40, 1.50)	
Other	39/96 (40.6%)	28/81(34.6%)	0.41	
Donor type				
Matched (sibling/unrelated)	90/98(91.8%)	79/86 (92.9%)	0.99(0.29, 3.30)	
Mismatched (haploidentical, unrelated)	8/98(8.2%)	7/86(7.1%)	1	
Conditioning				
Myeloablative	49/98(50%)	56/86(65.1%)	0.53(0.28, 1.01)	0.98(0.41, 2.37)
Reduced intensity	49/98(50%)	30/86(34.9%)	0.04	*P* = 0.98
Total body irradiation				
Yes	34/98 (34.7%)	28/86(32.6%)	1.1 (0.57, 2.13)	**2.35(0.99, 5.58)**
No	64/98(65.3%	58/86(67.4%)	0.76	*P* ** = 0.052**
GVHD				
Yes	61/96(63.5%)	60/85(70.6%)	0.73(0.37, 1.42)	
No	35/96(36.5%)	25/85(29.4%)	0.31	
Medications				
Immunosuppression				
Yes	22/98 (22.5%)	24/86(27.9%)	0.75(0.36, 1.54)	
No	76/98(77.5%)	62/86(72.1%)	0.39	
DASS 21 score (median, IQR**)**	19(8,30)	20(8,40)		*P* = 0.36
Depression subscale	4(0,8)	6(2,12)		*P* ** = 0.04**
Anxiety subscale	4(2,8)	4(2,10)		*P* = 0.3
Stress subscale	10(3,14)	8(2,16)		*P* = 0.99
FACT‐BMT score (Median, IQR)	114(101, 126)	107(93, 119)		*P* ** = 0.02**
Physical well‐being subscale	25(21,27)	24(20,27)		*P* = 0.24
Social well‐being subscale	22(17,26)	20(17,25)		*P* = 0.18
Emotional well‐being subscale	18(16,19)	16(13,18)		*P* ** < 0.001**
Functional well‐being subscale	21(17,26)	20(16, 23)		*P* = 0.15
BMT well‐being subscale	29(25,32)	27(23,31)		*P* = 0.049
LEE GVHD score (Median, IQR)	12(7, 24)	19(11, 29)		*P* = 0.02
Global severity GVHD symptoms (%)				
Mild	12(16.0%)	6 (17.1%)		*P* = 0.68
Moderate	41 (54.7%)	15 (42.9%)		
Severe	19 (25.3%)	12 (34.3%)		
Very Severe	3(4.0%)	2(5.7%)		
Fear of cancer recurrence (Median, IQR)	13(9, 15)	14(9, 17)		*P* = 0.48
Post transplant growth inventory score	61(51, 75)	59(42, 72)		*P* = 0.26

AUD, Australian Dollars; BMT, Bone marrow transplantation; IRQ, interquartile ranges; PTGI, The Post Traumatic Growth Inventory

a Potential confounders included in multivariable logistic regression: age, residential status, marital status, early post transplant (within 2 years), conditioning regimen (myeloablative compared to reduced intensity), and whether total body irradiation was used (Yes, No).

Bold text indicates statistically significant figures.
